# Incidence and Severity of Prescribing Errors in Parenteral Nutrition for Pediatric Inpatients at a Neonatal and Pediatric Intensive Care Unit

**DOI:** 10.3389/fped.2017.00149

**Published:** 2017-06-30

**Authors:** Theresa Hermanspann, Mark Schoberer, Eva Robel-Tillig, Christoph Härtel, Rangmar Goelz, Thorsten Orlikowsky, Albrecht Eisert

**Affiliations:** ^1^Hospital Pharmacy, RWTH Aachen University Hospital, Aachen, Germany; ^2^Department of Pediatric and Adolescent Medicine, Section of Neonatology, RWTH Aachen University Hospital, Aachen, Germany; ^3^Department of Neonatology, St. Georg Hospital, Leipzig, Germany; ^4^Department of Pediatric and Adolescent Medicine, University Hospital Schleswig-Holstein, Lübeck, Germany; ^5^Department of Neonatology, University Children’s Hospital Tuebingen, Tuebingen, Germany

**Keywords:** medication error, medication safety, child, neonate, intensive care unit, order error, clinical significance

## Abstract

**Objectives:**

Pediatric inpatients are particularly vulnerable to medication errors (MEs), especially in highly individualized preparations like parenteral nutrition (PN). Aside from prescribing *via* a computerized physician order entry system (CPOE), we evaluated the effect of cross-checking by a clinical pharmacist to prevent harm from PN order errors in a neonatal and pediatric intensive care unit (NICU/PICU).

**Methods:**

The incidence of prescribing errors in PN in a tertiary level NICU/PICU was surveyed prospectively between March 2012 and July 2013 (*n* = 3,012 orders). A pharmacist cross-checked all PN orders prior to preparation. Errors were assigned to seven different error-type categories. Three independent experts from different academic tertiary level NICUs judged the severity of each error according to the National Coordinating Council for Medication Error Reporting and Prevention (NCC MERP) Index (categories A–I).

**Results:**

The error rate was 3.9% for all 3,012 orders (118 prescribing errors in 111 orders). 77 (6.0%, 1,277 orders) errors occurred in the category concentration range, all concerning a relative overdose of calcium gluconate for peripheral infusion. The majority of all events (60%) were assigned to categories C and D (without major harmful consequences) while 28% could not be assigned due to missing majority decision. Potential harmful consequences requiring interventions (category E) could have occurred in 12% of assessments.

**Conclusion:**

Next to systematic application of clinical guidelines and prescribing *via* CPOE, order review by a clinical pharmacist is still required to effectively reduce MEs and thus to prevent minor and major adverse drug events with the aim to enhance medication safety.

## Introduction

Pediatric inpatients, especially neonates and premature infants are at a high risk of medication errors (MEs) (1–6). This is caused by the high proportion of individually dosed, compounded drugs that are often not licensed for this age group (7). Parenteral nutrition (PN) takes an important part in drug therapy in the pediatric and neonatal intensive care unit (PICU/NICU) (8). Early nutritional support improves growth and neurodevelopmental outcome (9, 10). PN is needed, as long as enteral fluid and energy intake can not cover the patients’ demand (11). PN composition requires accurate calculations of fluid and energetic content, macro- and micro-nutrients, total osmolarity, and observance of compatibility. The large number of individual calculation steps in each new order accounts for a high probability of error (12). The Institute for Safe Medication Practices lists PN preparations as a class of high alert medications (13). They pose a notably high risk of causing patient harm when used in error (14). Errors may occur during any stage of the medication process, but they are especially prevalent during the prescribing process (15, 16). Published guidelines provide support in this process (8, 17, 18). Computerized physician order entry (CPOE) systems are designed to reduce prescribing errors (16, 19, 20). In addition, clinical pharmacists play an important role in reducing MEs in pediatric patients (21–24).

The purpose of this study was to determine the incidence, type, and severity of errors in pediatric PN orders in our institution, to explore which errors a clinical pharmacist can reduce parallel to a CPOE system with the aim to optimize quality and safety in our PN prescribing process.

## Materials and Methods

### Setting

The study was conducted at the RWTH Aachen University Hospital and has been approved by the local ethics committee of the Medical faculty of RWTH Aachen University (EK 126/13).

The type, incidence, and severity of prescribing errors in PN in a tertiary level (single center) NICU/PICU of 18 beds (all equipped for mechanical ventilation) were surveyed prospectively for all PN orders between March 2012 and July 2013 (*n* = 3,012) by a clinical pharmacist during his daily routine check. Time period was selected related to the presence of the clinical pharmacist. According to the current Federal German pharmacy work rules, there is a requirement that a pharmacist will review the orders of individual formulations like PN before preparation. In our institution, this reviewing is performed by or under direct supervision of a clinical pharmacist. Clinical pharmacists have undergone a 3-year postgraduate specialization and are examined by the German chamber of pharmacists.

### Data Collection

Neonatal and pediatric intensive care unit PN orders in our institution are regularly performed using a CPOE system (Visite 2000, Lyomark Pharma GmbH, Oberhaching, Germany). Visite 2000 was introduced in November 2007. It had been established in prescription practice for over 4 years prior to the beginning of this study. Decision support is provided by calculating the total intake of fluids, nutrients, and electrolytes during 24-h period. This takes into account all medication as well as enteral and PN, which has been prescribed based on patient age and weight. Standards for PN formulations and notifications for drug dosing for different patient ages are integrated. Alerts for drug dosing (amino acids, trace elements, electrolytes, and fluids) as well as osmolarity were already integrated in the software upon its introduction and maintenance. Warnings for exceeding osmolarity occur only after the selection of peripheral vein administration by the physician. Warnings for incompatibility or drug–drug interactions are not features of Visite 2000. Alerts for calcium overdose for peripheral vein administration had not been included into the database prior to the beginning of this study.

After approval through the physician, the pharmacist has online access to the CPOE and performs order review. This process comprises the verification of patient data, ingredients, appropriate dose according to age, weight, and clinical condition, adherence to maximum osmolarity for route of administration, as well as compatibility, stability, and completeness prior to preparation. The pharmacist discussed each deviation from standard with the responsible physician and eliminated errors before preparing PN.

The MEs were defined according to the US National Coordinating Council for Medication Error Reporting and Prevention (NCC MERP) as “any preventable event that may cause or lead to inappropriate medication use or patient harm while the medication is in the control of the health care professional, patient, or consumer. Such events may be related to professional practice, health-care products, procedures, and systems, including prescribing, order communication, product labeling, packaging, and nomenclature, compounding, dispensing, distribution, administration, education, monitoring, and use” (25). Therefore, a prescribing error in our study was determined as deviation from a “defined standard,” not intended from the physician that may result in patient harm. “Defined standard” meant orders that were not in accordance with the manufacturer’s summary of product characteristics, local PN-guidelines, as well as national (26) or international consensus guidelines (8, 18). Our hospital guidelines are based on these consensus guidelines and were only used as reference where no recommendation was given in consensus guidelines. This refers especially to calcium concentrations in PN prescriptions for peripheral vein administered infusions for which no standard is given in the guidelines mentioned above.

In a first step after study inclusion, the pharmacist categorized the MEs into seven different types of error: patient data, drug choice, dosage, indication, compatibility, concentration range, and osmolarity (Table 1). In the categories dosage and concentration range, only deviations, which exceeded a 5% tolerance range from the defined standard were counted as ME. We selected a tolerance of 5% because it is a conventional pharmaceutical practice in the preparation of pharmaceuticals. We calculated the overall error rate as the percentage of errors relative to total drug orders and the error rate of each category relative to drug orders according to the category with 95% confidence intervals (CIs). The calculation of CI was performed with Microsoft^®^ Excel^®^, Version 14.3.0, Microsoft Corporation, Redmont, Washington, USA.

**Table 1:** Error types and definitions.

Error category	Definition
Patient data	Wrong patient name, date of birth, or weight
Drug choice	Prescription of wrong component in parenteral nutrition (PN). This included a wrong choice of drug concentration (e.g., 5% glucose instead of 50% glucose)
Dosage	Wrong dosage of a drug in PN. Drug dosage is specified on a daily basis adjusted to body weight
Indication	Prescription of a drug without indication, or omission of a drug even though the drug was indicated
Compatibility	PN mixture that may be chemically incompatible. The lack of amino acids in PN is regarded as a potential cause of a precipitation of calcium phosphate in solutions simultaneously containing calcium gluconate and sodium glycerophosphate. Equally, the lack of amino acids in PN exposes water-soluble vitamins to an increased degradation through trace elements
Concentration range	Wrong concentration range in PN, especially a concentration range of calcium gluconate above 0.4% in PN that is applied *via* a peripherally inserted venous catheter
Osmolarity	Osmolarity of a PN that is applied over a peripherally inserted venous catheter should be lower than 750 mosmol/l. Osmolarity from 750 mosmol/l to 800 mosmol/l is accepted. An error is defined as an osmolarity above 800 mosmol/l.

**Figure 1:**
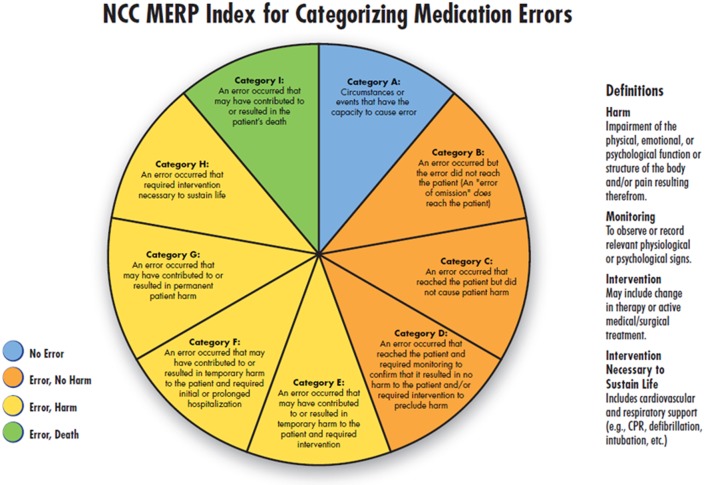
National Coordinating Council for Medication Error Reporting and Prevention (NCC MERP) index for categorizing medication errors. © 2001 National Coordinating Council for Medication Error Reporting and Prevention. All Rights Reserved. Permission is hereby granted to reproduce information contained herein provided that such reproduction shall not modify the text and shall include the copyright notice appearing on the pages from which it was copied.

In a second step, the potential severity of each ME was assessed independently by three experts from three different tertiary level NICUs under the hypothesis that the ME had reached the patient. The experts were senior neonatologists not affiliated with our institution and not involved in the development of our institutional guidelines. None of the experts was involved in any therapy regime, and all were blinded for patients and their outcome. Severity classification was based on the NCC MERP index (27) (Figure 1). For the expert classification, every prescribing error was described anonymously including patient data (age and weight), medication, and recent laboratory results. Expert training was carried out *via* a standard operating procedure that includes instructions how to use the NCC MERP index while predicting severity. The experts had the choice between the NCC MERP index categories C, D, E, F, G, H, and I. Categories A and B were excluded according to the hypothesis under which the assessment was done. A consensus was reached if all experts voted for the same level. If a minimum of two experts came to the same result, a “majority decision” was achieved. If there was no consensus or majority decision, errors dropped out of analysis but are discussed individually in the results section and discussion.

## Results

118 prescribing errors were found in 111 out of 3,012 orders (error rate 3.9%; 95% CI 3.2–4.6).

### Error Categories

**Figure 2:**
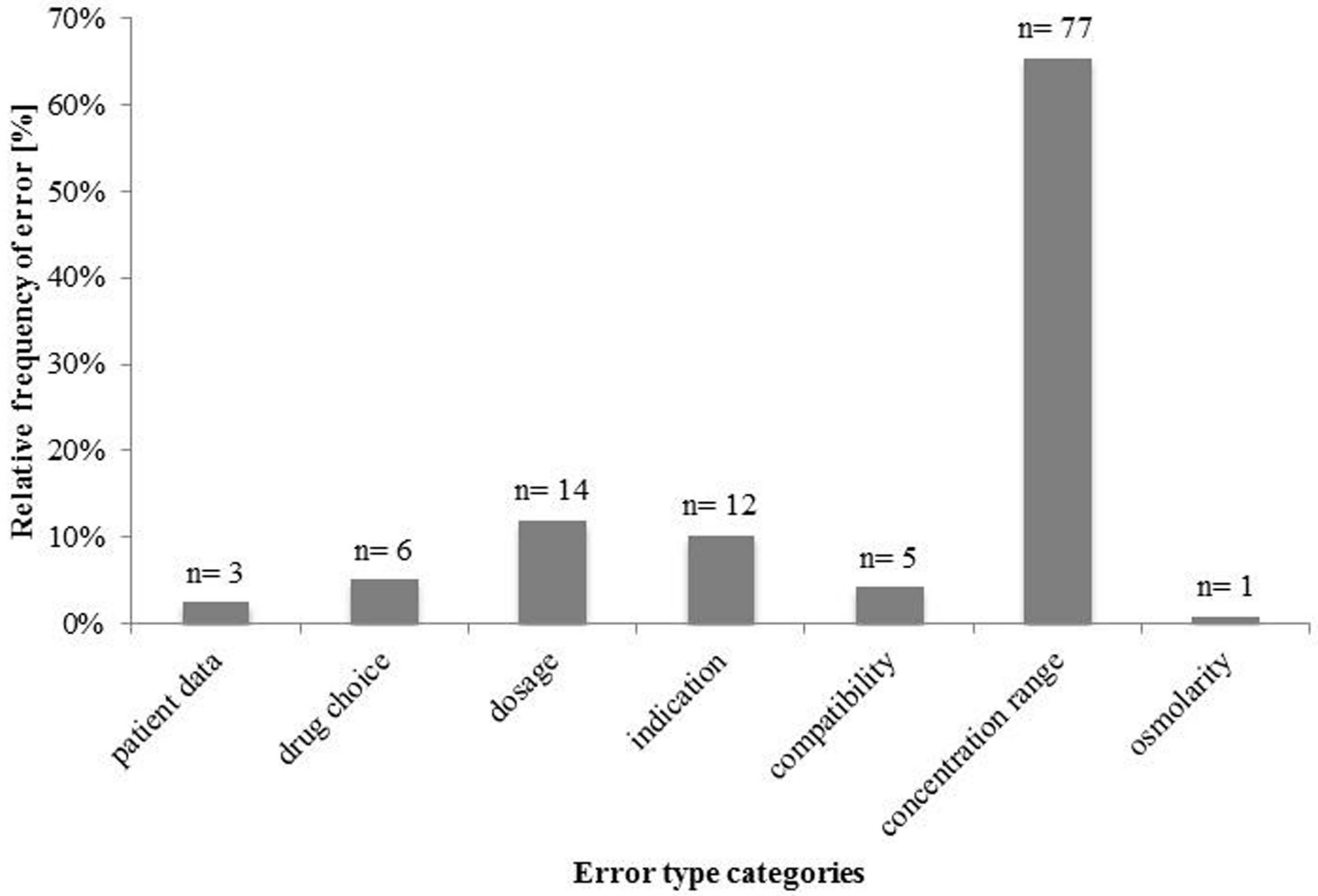
Incidence and type of errors of prescribing parenteral nutrition (*n* = 118).

The pharmacist classified the errors into the seven error-type categories patient data, drug choice, dosage, indication, compatibility, concentration range, and osmolarity (Figure 2).

Most errors were assigned to the category “concentration range” (*n* = 77 in 1,277 orders, 6.0% error rate; 95% CI 4.7–7.3). The error rate was calculated relative to the drug orders with calcium gluconate and for peripheral vein administration. All of these were calcium concentrations above 0.4% in solutions intended for peripheral vein infusion. The concentrations of calcium gluconate in these PN solutions ranged from 0.42 to 1.71% (see Table S1 in Supplementary Material). There were 14 dosing errors in 3,012 orders, giving an error rate of 0.46%; 95% CI 0.22–0.71. Most “dosage” errors (*n* = 12) were related to wrong dosages of water-soluble vitamins and trace elements in PN (Table 2), most of which would have led to errors of less than 10% increase from the accepted range. One error would have led to an overdose of potassium chloride of 43%, another one would have caused an overdose of sodium chloride of 26% (Table 2). The third most frequent error-type category was “indication” errors (*n* = 12 in 3,012 orders, 0.40% error rate; 95% CI 0.17–0.62). All errors would have led to an omission of a PN component even though this component was indicated. Six (50%) errors resulted from the omission of water-soluble vitamins, five (42%) errors from omission of trace elements, and one (8%) from omission of calcium gluconate. Six errors in 3,012 orders (0.20%; 95% CI 0.04–0.36) were assigned to the “drug choice” error category. In five of these cases, the wrong concentration of glucose was prescribed. The standard for preparing PN is a 50% glucose solution. The physician prescribed the correct dose of glucose but chose the wrong concentration (5% instead of 50%). Inadvertent order of 5.85% sodium chloride instead of 20% sodium chloride solution, the standard in pharmacy PN compounding, occurred once. Five errors in 1,898 orders (0.26%; 95% CI 0.03–0.49) were assigned to the error-type category “compatibility.” The error rate was calculated relative to the drug orders with simultaneously prescribing of calcium gluconate and sodium glycerophosphate or trace elements and water-soluble vitamins. These formulations would have contained no amino acids and, therefore, would have been chemically unstable. In three instances, this was due to the simultaneous order of sodium glycerophosphate and calcium gluconate and twice due to the simultaneous order of water-soluble vitamins and trace elements. The category “patient data” contained three events (0.1%). In each instance, a wrong patient age was conveyed to the pharmacy. The single event (0.06%) in the error-type category “osmolarity” was an excess osmolarity (933.7 mmol/l) intended for a peripheral vein administration. Due to the small number of errors in the categories patient data and osmolarity, the 95% CIs were not calculated.

**Table 2:** Incidence and type of dosage errors (12%, *n* = 14).

Type and percentage of dosage error	Wrong dosage^a^	Correct dosage^a^	Deviation (%)
Underdose of trace elements (43%)	0.76 ml/kg/day	0.91 ml/kg/day	17
	0.71 ml/kg/day	1.0 ml/kg/day	29
	0.92 ml/kg/day	1.0 ml/kg/day	8
	0.92 ml/kg/day	1.0 ml/kg/day	8
	0.92 ml/kg/day	1.0 ml/kg/day	8
	0.92 ml/kg/day	1.0 ml/kg/day	8
Overdose of water-soluble vitamins (36%)	1.05 ml/kg/day	1.0 ml/kg/day	5
	1.08 ml/kg/day	1.0 ml/kg/day	8
	1.08 ml/kg/day	1.0 ml/kg/day	8
	1.08 ml/kg/day	1.0 ml/kg/day	8
	1.08 ml/kg/day	1.0 ml/kg/day	8
Overdose of potassium chloride 7.45% (7%)	2.86 mmol/kg/day	2.0 mmol/kg/day	43
Overdose of sodium chloride 20% (7%)	5.57 mmol/kg/day	4.43 mmol/kg/day	26
Underdose of water-soluble vitamins (7%)	0.57 ml/kg/day	1.0 ml/kg/day	43

*^a^Body weight-related dosage*.

### Classification of Clinical Significance (NCC MERP)

**Figure 3:**
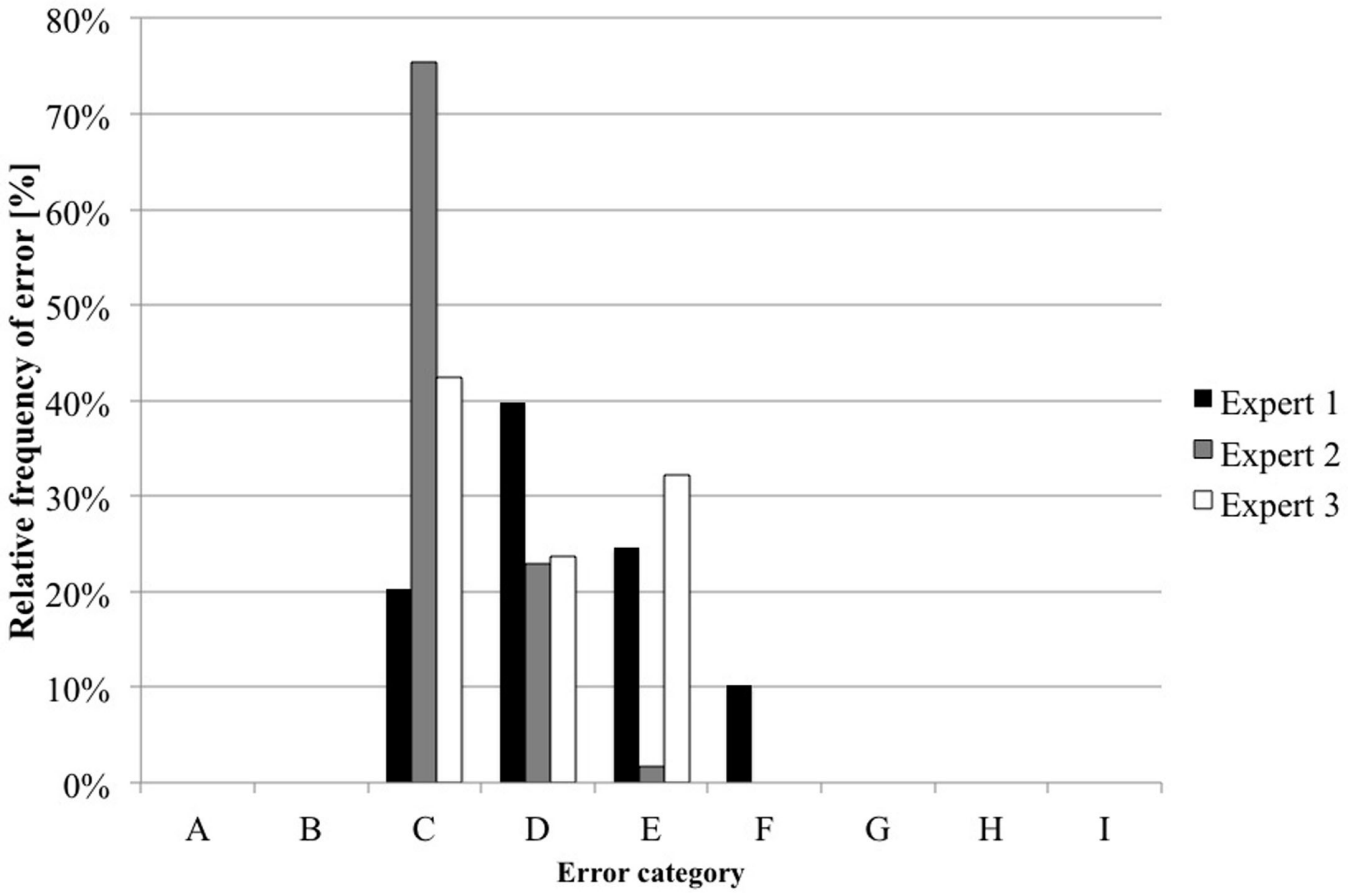
Results of error classification by the three experts (*n* = 3*118). Expert 1 failed to classify six (5%) errors; expert 3 did not classify two (2%) errors.

The three experts classified the 118 errors independently according to the NCC MERP index (Figure 3). In eight instances, errors were not classified unintentionally by two experts and, therefore, they are not presented in the chart.

One expert did not classify one error of the category drug choice (the wrong concentration of glucose was prescribed) and one error of the category dosage (underdose of trace elements were prescribed, 0.71 ml/kg/day instead of 1 ml/kg/day).

The other expert did not classify one error of the category drug choice (the wrong concentration of glucose was prescribed), three errors of the category concentration range (calcium gluconate 0.5, 0.65, and 0.46% instead of the maximum 0.4%), and two errors of the category dosage (underdose of trace elements were prescribed, 0.71 ml/kg/day instead of 1 ml/kg/day, same error as the other expert and underdose of trace elements 0.92 ml/kg/day instead of 1 ml/kg/day) (see Table S2 in Supplementary Material).

**Figure 4:**
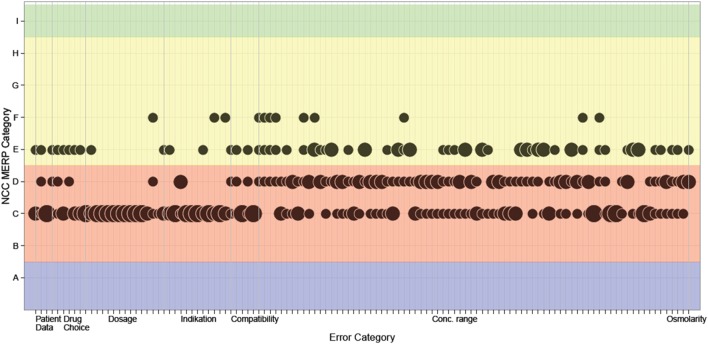
Results of classification of clinical significance. The classification of each event by each expert is depicted in the figure. If more than one classification fell into the same National Coordinating Council for Medication Error Reporting and Prevention (NCC MERP) category (*y*-axis), this is indicated by bubble size. Corridors on the *x*-axis mark error type categories.

Majority decisions were found for 85 (72%) errors. In 23 (20%) of these errors, the three experts found consensus, all concerning category C. Most of events (38%, *n* = 45) were categorized into category C, 22% (*n* = 26) into category D, and 12% (*n* = 14) in category E. In 27 (23%) errors, the assessment by the three experts was spread between three NCC MERP categories. Five errors were classified only by two experts into two different categories; one error was classified only by one expert. The variability between the experts while assessing the prescribing errors is shown in Figure 4.

In the category “concentration range,” an agreement of all three experts was reached in four errors, all concerning category C. For the remaining 73 errors, the assessment of the experts varied between C and D (34 errors), C, D, and E (14 errors), C and E (5 errors), D and E (11 errors), D, E, and F (8 errors), and E and F (one error). In the category “dosage,” ten errors, all relating to a wrong dosage of water-soluble vitamins or trace elements, were assigned to category C by all experts. The error related to an overdose of sodium chloride was categorized into C and E and the one related to an overdose of potassium chloride was assigned to C, D, and F. Six errors in the category “indication,” all related to the omission of water-soluble vitamins, were assigned to category C by all experts. The six remaining errors fell in category C and D (omission of calcium gluconate), C and E or C and F (both omission of trace elements). The errors in the category “drug choice” were assessed differently. While one expert classified nearly all errors into category E, the two other experts assigned most of the six errors to category C or D. For three errors, a majority decision for category C was reached. In the category “compatibility,” the two errors “simultaneous prescribing of water soluble vitamins and trace elements in the absence of amino acids” (40%, *n* = 2), were classified by all three experts into category C. For the three remaining errors, “simultaneous prescribing of sodium glycerophosphate and calcium gluconate in the absence of amino acids” (60%, *n* = 3), the assessment of the experts varied between C, D, and E. In the category “patient data,” an agreement of all three experts was found for one error (category C) while for two errors the assessment differed between the categories C and E. The error categorized in C, D, and E resulted from an order with a patient age of 28 years although the patient was 1-day old. The error categorized into C and E was an order with a patient age of 11 weeks although the patient was 2 years and 11 weeks old. The error in the category “osmolarity” was assigned for majority decision to category D and from one expert to category E.

## Discussion

Medication errors in PN can occur during prescribing, transcription, preparation, and administration. We focused on the incidence of errors during the prescribing process of PN for pediatric inpatients. The published data regarding prescribing errors in pediatric PN are scant. The study of Sacks et al. (28) included pediatric and adult patients and examined the entire medication process. Errors and adverse events were entered into a real-time web-based database and then classified into harm scores depending on the severity of the adverse event based on the NCC MERP Index. Most errors occurred during transcription and administration (28). MacKay et al. conducted a similar study in a children’s hospital. Most errors arose during administration (29). Both studies reported a rate of MEs related to the prescription, transcription, preparation and administration of PN of only 6% (28) and 0.27% (29). In contrast to our study, the errors observed in the study of Sacks et al., excluding the prescribing errors, were not detected before preparation. In both studies the severity of errors was classified based on the clinical outcome of the actual adverse events. The design of our study focuses on errors on the stage of prescription for which adverse events were avoided, because a clinical pharmacist detected and corrected the errors before preparation.

Other studies were in the same way limited to PN prescribing errors. Brown et al. reduced the neonatal PN prescribing error rate by introducing a computerized PN worksheet from 14.5 to 6.8% (16); Lehmann et al. reduced the error rate from 10.8 to 4.2% and 1.2% in a NICU in two intervention periods by introducing an online total PN order entry system (30). In the study of Hilmas and Peoples, a clinical pharmacist in a pediatric hospital had to intervene in 5% of PN orders (31). As in our study a clinical pharmacist prevented adverse drug events. In summary, the overall incidence of prescribing errors in our study (3.9%, *n* = 118) is in line with the published data regarding only prescribing errors in PN. The studies discussed above used different error type classifications. Brown et al. only defined three prescribing error types (volume error, calcium/phosphate solubility error, osmolarity error) (16). In contrast to our study, MacKay et al. assigned compatibility errors to the administration process, not to prescribing (29).

In our study, the classification of severity of the prescribing errors by the three experts was rather variable. One reason is the difficulty to value the hypothetical impact of errors that did not reach the patient. The experts assessed the errors on the basis of their own experiences. Heterogeneity of expert experiences is due to different hospital standards and due to a lack of national guidelines. Consensus was found mostly for errors that were regarded as less serious.

The majority of errors were assigned to the categories C and D (“error reached the patient, no harm,” 60%). Classification in category E (“error reached the patient, temporary harm”) was chosen in 12% of assessments. In the study of Sacks et al., six (8%) of the 74 errors contributed to or resulted in temporary harm to the patient (28). In the study of MacKay et al., 21 (9.1%) of 230 errors were classified as to have potentially contributed to temporary harm (category E, F) (29).

In our study, the category with the highest number of errors was the category “concentration range.” All errors in this category were attributable to orders of a calcium gluconate concentration higher than 0.4% in PN intended for application *via* peripherally inserted venous catheters. Increased deviation of the “standard” resulted in more “serious” categories. This is based on the assumption that the amount of calcium in PN given *via* a peripheral venous catheter has to be restricted (32, 33). International guidelines indicate the risk of calcium in parenteral solutions to cause damage to peripheral veins and extravasation may induce severe tissue necrosis (8). Since the demand for calcium and phosphorus especially in preterm infants exceeds the amount that is assumed to be tissue-compatible with peripheral infusion, physicians aim to meet the upper limit of the tolerable range. Moreover, the safe concentration is not quite clear, since there is a lack of references about this topic. The range of calcium gluconate in PN in our study was determined by our own institutional guidelines and is a value of experience. Before revision of the guidelines in 2009, the upper limit of calcium concentration was higher (0.6%). This “historical” permissible value could be an additional explanation for exceeding the calcium concentration range of 0.4% by the physicians. The reason for the high variability of the three experts vote in this category could result from different standards of calcium limits in their institutions. Further studies are needed to determine the upper concentration range of calcium in PN with an acceptable risk of thrombophlebitis and necrosis, still ensuring a sufficient supply for newborns with calcium. While the second major error category “dosage” was responsible for 11.9% of errors in our study, 39% (*n* = 139) of the interventions by the clinical pharmacist in the study of Hilmas and Peoples were related to errors of dosage and composition (31). Of all events, 6% were errors that could have resulted in an adverse drug reaction, medication error, or toxicity. Most (71%, *n* = 10) dosage errors in our study were assigned with expert consensus to category C, while the assessment of two (14%) errors varied between category C and E (wrong dosage of sodium chloride), respectively C, D, and F (wrong dosage of potassium chloride). Special attention is paid to this type of error, since potassium overdoses have a high potential for harm or even fatality (28, 34). The prescribed dose in our study was 2.89 mmol/kg/day, which exceeds the institutional guideline limit of 2 mmol/kg/day and was rated as an error because it was prescribed unintentionally. It is remarkable that only one error was related to an overdose of potassium chloride in our study, while other studies showed that potassium chloride is more often associated with errors (15). The four errors because of an overdose of soluble vitamins (1.08 ml/kg/day instead of 1.0 ml/kg/day) and the four errors referring to an underdose of trace elements (0.92 ml/kg/day instead of 1.0 ml/kg/day) resulted from a systematic rounding error in our CPOE. With the next generation CPOE Visite 2015, this type of error will be eliminated. Similar results can be found in the category “compatibility.” While three errors were classified with consensus in category C, the three errors “simultaneous prescribing of sodium glycerophosphate and calcium gluconate in the absence of amino acids” were classified with considerable variability (C, D, and E). In the study of MacKay et al., 13% of the administration errors were due to incompatibility. One of these errors resulted in temporary harm (29).

In our study, none of the described errors reached the patient because a clinical pharmacist checked and discussed the orders with the physician before compounding. An additional alert in the computerized physician order entry (CPOE) software regarding the upper calcium concentration limit was installed as a consequence of this study to reduce the most frequent error type in the category concentration range. Furthermore, we are looking for a change in the next generation CPOE that is less prone to rounding errors. Nevertheless, if a CPOE is equipped with all possible warnings, the risk for over alerting is high, and the user might ignore the notifications. Additionally, the available CPOEs often do not sufficiently represent the requirements for pediatric (especially neonatal) drug orders. Studies have shown that although CPOE systems often decrease the prescribing error rate, they can also lead to other types of errors at the same time (35, 36). In the study by Jani et al., for example, the physician chose a wrong dosing interval from a CPOE a drop-down menu (36).

Therefore, due to complexity of prescribing PN and the diversity in the pediatric patient group, it is necessary to have a clinical pharmacist review the final PN orders. The clinical pharmacist may then detect errors, which the CPOE failed to prevent, or those, which it may have caused.

### Limitations

One limitation of this study is that we only studied the situation of prescribing PN in one department of our hospital. The results are difficult to generalize because CPOEs, standards, guidelines, and methods to categorize MEs may differ depending on institutional policies. Another limitation is that only one pharmacist conducted the analysis, which may cause observation bias. Therefore, the classification of error severity was delegated to three independent experts who are unaffiliated with our institution.

While MEs can occur at all stages of the medication process, we focused on prescribing errors. To enhance the medication safety in the whole PN process, further studies are needed to analyze errors in the transcription, preparation, and administration of PN.

Additionally, we did not address errors that might have been missed by the pharmacist. But as we observed no clinically overt complications or metabolic derangements related with TPN in the study period, we assume that there was at least no missed error of clinical significance.

We used the NCC MERP index to categorize our prescribing errors. The index was developed to classify MEs on the basis of patient outcome. However, the classification in our study was based on the estimated potential of harm to the patient, not on actual outcome. Additionally, the experts assessed the errors independently without interaction among each other. An error assessment of an expert group could have resulted in a different outcome. Expert classification of the relevance of errors in this study has shown a larger variability than expected. All experts were provided the same standard operating procedure for the judgment of the clinical relevance of errors. It was requested to categorize the errors on the assumption that the “worst possible event” had occurred. We believe that this is most likely the explanation for the high variability. So, despite the identical specification for all experts, it is to some degree left to the individual imagination of each expert, which would be the most extreme but still realistic combination of unfavorable circumstances. We chose to assume the worst case, because it is most significant for the individual patient security and should most certainly be avoided. However, judgment of the clinical relevance, which is to be expected most commonly based on the clinical experience of the experts might have resulted in a more homogenous classification of events.

### Conclusion

Parenteral nutrition order in pediatric inpatients is highly individualized and includes a large number of calculations and characteristics to respect. It is, therefore, particularly prone to errors. However, only a small percentage of errors carries the risk to result in patient harm. If physicians and pharmacists are aware of these risks, measures can be developed to avoid errors. Above and beyond an alert system in the CPOE, a clinical pharmacist is proven to be very effective to improve medication safety in a medical team and to prevent adverse drug events.

## Author Contributions

TH designed and conducted the analysis. She analyzed the data and drafted the first manuscript. MS designed and assisted with the analysis and corrected the manuscript. ER-T, CH, and RG interpreted the data while assessing the clinical significance and reviewed the manuscript. TO and AE contributed to the conception of the analysis and reviewed the manuscript. Each author listed on the manuscript has seen and approved the final manuscript as submitted and takes full responsibility for the manuscript. All authors are accountable of all aspects of the work in ensuring that questions related to the accuracy or integrity of any part of the work are appropriately investigated and resolved.

## Conflict of Interest Statement

The authors declare that the research was conducted in the absence of any commercial or financial relationships that could be construed as a potential conflict of interest.

## Abbreviations

CIs, confidence intervals; CPOE, computerized physician order entry; ME, medication error; NCC MERP, National Coordinating Council for Medication Error Reporting and Prevention; NICU, neonatal intensive care unit; PICU, pediatric intensive care unit; PN, parenteral nutrition.
