# Supplementary material for: Incidence and Severity of Prescribing Errors in Parenteral Nutrition for Pediatric Inpatients at a Neonatal and Pediatric Intensive Care Unit
Source: Front Pediatr. 2017 Jun 30;5:149. doi: 10.3389/fped.2017.00149 (PMC5491912; doi:10.3389/fped.2017.00149)
Supplement: Supplementary file 1 [file Table_1.DOCX]

Table S1

Extent and severity of errors of the category concentration range: Calcium gluconate concentration above 0.4 % in PN that is applied via a peripherally inserted venous catheter.

| Concentration range of calcium gluconate  above 0.4 % | Results of majority decision of expert classification according to the NCC MERP Index^a^ |
| --- | --- |
| 0.42 | C |
| 0.42 | C |
| 0.42 | C |
| 0.42 | No majority decision |
| 0.43 | C |
| 0.43 | C |
| 0.43 | C |
| 0.43 | C |
| 0.43 | No majority decision |
| 0.43 | No majority decision |
| 0.43 | No majority decision |
| 0.44 | C |
| 0.44 | C |
| 0.44 | C |
| 0.45 | D |
| 0.45 | D |
| 0.45 | D |
| 0.45 | C |
| 0.46 | D |
| 0.46 | D |
| 0.46 | D |
| 0.46 | D |
| 0.46 | D |
| 0.46 | D |
| 0.46 | D |
| 0.46 | No majority decision |
| 0.47 | D |
| 0.47 | D |
| 0.47 | D |
| 0.47 | D |
| 0.47 | D |
| 0.48 | D |
| 0.48 | D |
| 0.48 | D |
| 0.48 | D |
| 0.48 | D |
| 0.48 | D |
| 0.49 | D |
| 0.50 | No majority decision |
| 0.50 | No majority decision |
| 0.50 | No majority decision |
| 0.51 | No majority decision |
| 0.51 | No majority decision |
| 0.52 | No majority decision |
| 0.53 | No majority decision |
| 0.54 | E |
| 0.55 | No majority decision |
| 0.55 | No majority decision |
| 0.55 | E |
| 0.55 | No majority decision |
| 0.56 | No majority decision |
| 0.57 | E |
| 0.58 | E |
| 0.58 | No majority decision |
| 0.58 | No majority decision |
| 0.59 | E |
| 0.59 | No majority decision |
| 0.60 | E |
| 0.62 | E |
| 0.62 | E |
| 0.62 | D |
| 0.63 | E |
| 0.64 | No majority decision |
| 0.65 | E |
| 0.65 | No majority decision |
| 0.67 | D |
| 0.67 | E |
| 0.69 | No majority decision |
| 0.71 | No majority decision |
| 0.72 | No majority decision |
| 0.73 | No majority decision |
| 0.73 | E |
| 0.81 | E |
| 0.89 | No majority decision |
| 1.49 | No majority decision |
| 1.71 | E |
| 1.71 | E |

^a^National Coordinating Council for Medication Error Reporting and Prevention. NCC MERP Index for Categorizing Medication Errors (2001) [cited 2016 March 15]. Available from: <http://www.nccmerp.org/sites/default/files/indexColor2001-06-12.pdf>.
