# Supplementary material for: Incidence and Severity of Prescribing Errors in Parenteral Nutrition for Pediatric Inpatients at a Neonatal and Pediatric Intensive Care Unit
Source: Front Pediatr. 2017 Jun 30;5:149. doi: 10.3389/fped.2017.00149 (PMC5491912; doi:10.3389/fped.2017.00149)
Supplement: Supplementary file 2 [file Table_2.DOCX]

Table S2

Type of errors not classified by Expert 1 and 3

| Category | Type of error | Expert |
| --- | --- | --- |
| Drug choice | Prescription of glucose 5 % instead of glucose 50 % | Expert 1 |
| Drug choice | Prescription of glucose 10 % instead of glucose 50 % | Expert 3 |
| Dosage | Prescription of an underdose of trace elements: 0.71 ml/kg/d instead of 1 ml/kg/d | Expert 1, 3 |
| Dosage | Prescription of an underdose of trace elements: 0.92 ml/kg/d instead of 1 ml/kg/d | Expert 1 |
| Concentration range | Wrong concentration range of calcium gluconate: 0.46 % instead of the maximum 0.4 % | Expert 1 |
| Concentration range | Wrong concentration range of calcium gluconate: 0.50 % instead of the maximum 0.4 % | Expert 1 |
| Concentration range | Wrong concentration range of calcium gluconate: 0.65 % instead of the maximum 0.4 % | Expert 1 |
